# Unilateral electrical stimulation of mice induces transcriptional response in stimulated leg with limited effect on non‐stimulated contralateral leg

**DOI:** 10.1113/EP092394

**Published:** 2025-02-27

**Authors:** Takanaga Shirai, Kazuki Uemichi, Tohru Takemasa, Yu Kitaoka

**Affiliations:** ^1^ Department of Human Sciences Kanagawa University Yokohama Kanagawa Japan; ^2^ Research Fellow of Japan Society for Promotion Science Chiyoda‐ku Tokyo Japan; ^3^ Research Organization of Science and Technology Ritsumeikan University Kusatsu Shiga Japan; ^4^ Institute of Health and Sport Sciences University of Tsukuba Tsukuba Ibaraki Japan

**Keywords:** electrical stimulation, RNA‐seq, skeletal muscle, unilateral

## Abstract

Electrical stimulation is widely used to investigate localised muscle adaptations, with applications in both sports and rehabilitation. However, the systemic effects of electrical stimulation, particularly in the contralateral muscles that are not directly stimulated, are not well understood. This study investigated whether unilateral electrical stimulation induces transcriptional changes in both the electrically stimulated (ES) and non‐stimulated (non‐ES) contralateral legs, compared with the legs of sedentary control mice. RNA‐sequence analysis revealed that 1320 and 55 genes were differentially expressed in the ES and non‐ES, respectively, compared with controls using DEseq2 (false discovery rate cutoff = 0.05, minimal fold change = 1.5). Gene ontology and pathway enrichment analyses identified that the biological processes of immune response, muscle development, and response to stimuli were upregulated in the ES leg, while immune response and stress signalling were upregulated in the non‐ES leg. Although the non‐ES leg exhibited minimal transcriptional changes, *Tbc1d1*, which enhances glucose uptake, and *Mss51*, a regulator of mitochondrial function, were upregulated while *Ddit4*, a negative regulator of mammalian/mechanistic target of rapamycin signalling, and stress responsive protein *Gadd45g* were downregulated. These findings aid the understanding of molecular mechanisms underlying the cross‐education effect and suggest that contralateral effects of electrical stimulation are limited, despite potential signalling across the legs.

## Abstract (highlights)

- **What is the central question of this study?**
  Can unilateral electrical stimulation (ES) induce molecular adaptations in the contralateral, non‐stimulated leg, and what are the specific gene expression changes that potentially contribute to the cross‐education effect at the transcriptional level?
- **What is the main finding and its importance?**
  Unilateral ES primarily induces localised molecular adaptations in the stimulated muscle, but there are modest transcriptional changes in the contralateral non‐stimulated muscle, suggesting a limited systemic response. These findings highlight that while the primary effects of ES are localised, some cross‐leg molecular signalling may occur.

## 1 INTRODUCTION

Skeletal muscle exhibits significant plasticity, which adapts to a wide range of mechanical and metabolic stimuli (Abadi et al., 2009; Fiorenza et al., 2018), essential for muscle growth, recovery and performance optimisation. Such adaptations are relevant not only in the context of sports science but also in clinical settings, for example, rehabilitation following injury or bed rest (Schifino et al., 2023; Shur et al., 2024). Of interest is the cross‐education effect, the transfer of motor performance to the untrained (or untreated) contralateral homologous muscle (Manca et al., 2021). Although neural mechanisms are considered to underlie this effect (Carroll et al., 2006; Manca et al., 2018), the molecular basis remains less understood. Exploring the transcriptional adaptations underpinning cross‐education effects may provide further insights into how skeletal muscle responds systemically to localised stimuli, paving the way for new rehabilitation strategies.

Electrical stimulation (ES) is a well‐established method of inducing muscular contractions. It has been used extensively in both research and clinical applications for the study of muscular adaptations and the promotion of recovery (Behringer et al., 2018; Strasser et al., 2009; West et al., 2016; Yamada et al., 2019). Research investigating localised muscle adaptations have shown that ES can activate several molecular pathways linked to muscle growth and repair (Ogasawara et al., 2013). However, there is a lack of insight into the systemic effects of ES, particularly at the molecular level. Additionally, while ES has been used in unilateral models to study localised muscle adaptations, there has been little research into the transcriptional changes in the contralateral, unstimulated muscle.

Recent advancements in RNA‐sequencing have led to the development of powerful tools for exploring global transcriptomic changes in skeletal muscle (Cumming et al., 2024; Pataky et al., 2023). In this study, we applied this technology to a unilateral ES model in mice to investigate transcriptional changes in both stimulated and non‐stimulated legs. As repeated, transient increases in gene expression drives skeletal muscle adaptation, with different genes peaking at distinct time points following a stimulus (Kuang et al., 2022; Perry et al.,^2010^), muscle tissue samples were collected 3 h after the third ES session, which enabled the detection of both acute and sustained transcriptional responses. It was hypothesised that ES would also induce transcriptional changes in the non‐stimulated muscle.

## 2 METHODS

### 2.1 Ethical approval

All experimental procedures in this study were authorised by the Institutional Animal Experiment Committee of the University of Tsukuba (animal ethical permission number: 22‐397), based on the NIH *Guide for the Care and Use of Laboratory Animals* (NIH Publication, 1996).

### 2.2 Animals

Six male C57BL/6J (Tokyo Laboratory Animals Science Co., Tokyo, Japan) mice aged 7–8 weeks were used in this study, and randomly assigned to the two groups: mice receiving ES or not (*n* = 3 per group). The sample size was determined based on a previous study (Mori et al., 2021). The mice were kept in holding facilities under the following conditions: 22 ± 2°C temperature, 55 ± 5% humidity, 12 h light/dark cycle and ad libitum access to food and water. Upon completion of the experimental treatments, the mice were sacrificed via cervical dislocation. The gastrocnemius muscles were excised, weighed, quickly frozen in liquid nitrogen and stored at −80°C for further analyses.

### 2.3 Experimental design

**FIGURE 1:**
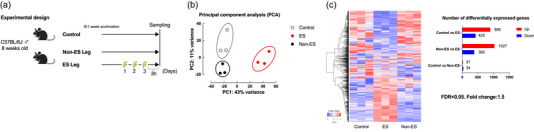
Effect of ES on gene expression in mice (*n* = 3 each group). (a) Experimental design. (b) Principal component analysis plot (*x*‐axis: PC1, *y*‐axis: PC2). (c) Heatmap and the number of differently expressed genes.

Figure 1a shows the experimental design. The ES protocol was performed under anaesthesia with inhaled isoflurane (2%, KN‐1701; Natsume, Tokyo, Japan) (Kitaoka et al., 2015; Shirai et al., 2022). Briefly, the mice were positioned with one foot on a footplate (with an ankle joint angle of 90°) in the prone position. The gastrocnemius muscle was stimulated percutaneously with electrodes connected to an electric stimulator and isolator (Ag/AgCl, Vitrode V; Nihon Kohden, Tokyo, Japan). The right gastrocnemius muscle was isometrically exercised (stimulation for 3 s, 10 contractions, with intervals of 7 s between contractions; total of five sets with 3‐min intervals between sets). The voltage (30 V) and stimulation frequency (100 Hz) were adjusted to produce the maximal isometric contraction torque (Kitaoka et al., 2015, 2016; Ogasawara et al., 2016). Electrodes were also attached to the left gastrocnemius muscle, but ES was not applied (non‐ES). ES was performed on three consecutive days, and muscle tissue was sampled 3 h after the end of the last set. The control mice underwent the same anaesthesia for consistency. Muscle tissues were frozen rapidly in liquid nitrogen and stored at −80°C until use.

### 2.4 RNA‐sequence and statistical analyses

After homogenisation in TRIzol reagent (Thermo Fisher Scientific, Waltham, MA, USA), the total RNA was extracted using the RNeasy Mini Kit (Qiagen, Hilden, Germany), and the RNA concentration was measured using the NanoDrop Lite (Thermo Fisher Scientific). Following verification of the sample quality using a 2100 Bioanalyzer (Agilent Technologies, Santa Clara, CA , USA), RNA‐seq was performed by Rhelixa Co. (Tokyo, Japan) as previously described (Kusano et al., 2024). Sequencing libraries were prepared using the NEBNext Poly(A) mRNA Magnetic Isolation Module (E7490) and an NEBNext UltraTMII Directional RNA Library Prep Kit (E7760, New England Biolabs, Ipswich, MA, USA), and 150 bp paired‐end reads were obtained using an Illumina NovaSeq 6000 (Illumina, Inc., San Diego, CA, USA). The quality of the raw sequencing reads was assessed with FastQC (Version 0.11.7). Low quality bases and adapter sequences were trimmed by Trimmomatic software (Version 0.38). The trimmed reads were aligned to the reference genome using HISAT2 (version 2.1.0), and the read counts were calculated using feature counts (version 1.6.3). All data were analysed using the integrated differential expression and pathway (iDEP) (http://bioinformatics.sdstate.edu/idep/) and GraphPad Prism software (v10.1.1, Macintosh, GraphPad Software, Boston, MA, USA). Raw read counts from each sample were normalised using the EdgeR algorithm (minimal counts per million = 0.5, pseudocount = 4, gene median). Enrichment of gene ontology (GO) was calculated with the gene ontology tool Database for Annotation, Visualisation and Integrated Discovery (DAVID). Differentially expressed genes (DEGs) were detected using DEseq2 (false discovery rate cutoff = 0.05, minimal fold change = 1.5).

## 3 RESULTS

**FIGURE 2:**
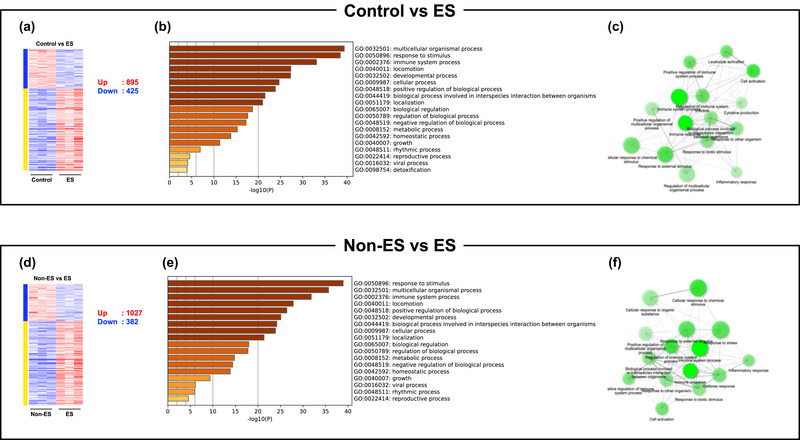
Gene expression profile and biological process between the two experimental groups. (a) Heatmap between the control and ES groups. (b) Bar plot of enriched GO terms. (c) Network diagram. (d) Heatmap between the non‐ES and ES groups. (e) Bar plot of enriched GO terms. (f) Network diagram.

**FIGURE 3:**
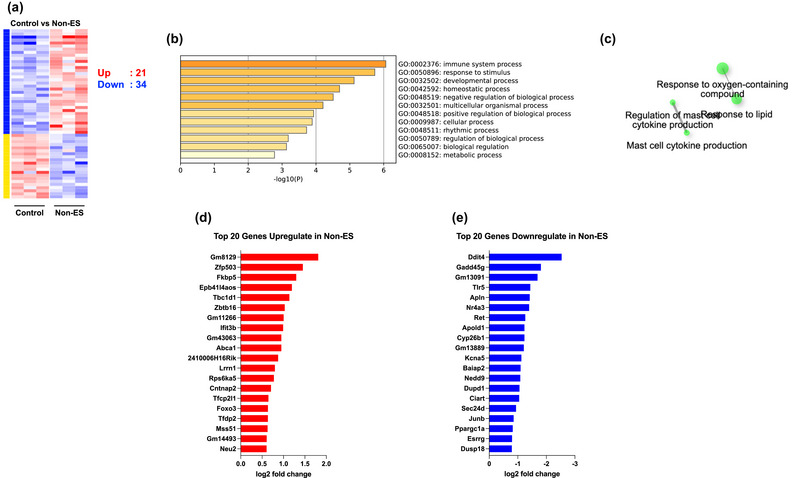
Comparison between the control and non‐ES groups. (a) Heatmap of differentially expressed genes. (b) GO enrichment analysis for DEGs. (c) Network diagram of the enrichment biological processes. (d) Top 20 upregulated genes in Non‐ES. (e) Top 20 downregulated genes in non‐ES.

Principal component analysis (PCA) was performed to visualise the variation in gene expression between groups (Figure 1b). While the plot shows clear segregation of the ES group from other groups, the non‐ES group also formed a distinct cluster from the baseline control group. The heatmap of DEGs across all groups shows clear clustering of gene expression profiles (Figure 1c). In the ES group, 895 genes were upregulated and 425 genes were downregulated versus the control group, while 1027 genes were upregulated and 382 genes were downregulated versus the non‐ES group. Of these DEGs after ES, 925 genes (716 increased and 209 decreased) were identical, indicating a similarity between the two comparisons. GO enrichment analysis revealed that the ES group exhibits significant enrichment of biological processes related to muscle contraction, oxidative phosphorylation, and metabolic pathways in comparison with the other two groups (Figure 2). Although not directly stimulated, the non‐ES group shows upregulation of 21 genes and downregulation of 34 genes compared with the control group; several genes are known to be exercise responsive genes (Figure 3). The top 20 upregulated genes, including *Tbc1d1* and *Mss51*, and the top 20 downregulated genes, including *Ddit4* and *Gadd45g*, are shown in Figure 3d and e, respectively. Supporting information Figure S1 shows the common and unique genes up‐ or downregulated in the ES group compared with the control and/or non‐ES groups, which highlights the transcriptional response in the muscle in the ES group.

## 4 DISCUSSION

This study investigated the molecular effects of unilateral ES on both the stimulated leg and the contralateral non‐ES leg, to identify if transcriptional changes in the non‐ES leg support the presence of a cross‐over effect. While significant transcriptional changes in the stimulated leg were observed, the responses in the non‐ES leg were minimal, which suggests that that contralateral effects of ES are limited.

The significant upregulation of genes associated with immune responses, muscle growth and developmental processes in the stimulated leg was consistent with previous studies, which suggested that ES triggered robust molecular adaptations, primarily driven by mechanical and metabolic stress on the stimulated muscle (West et al., 2016; Yamada et al., 2021). These findings support the well‐documented role of ES in the promotion of muscle hypertrophy and recovery through localised signalling pathways (Ogasawara et al., 2016; Yamada et al., 2019). The increase in muscle strength has been demonstrated following unilateral resistance exercise in the contralateral side (Carroll et al., 2006; Manca et al., 2018). However, our observation of the limited molecular response in the non‐ES leg raises important questions about the extent of the cross‐over effect at the transcriptional level. Previous research on unilateral exercise training in humans also indicates that the transient increase in muscle protein synthesis rate in the exercise leg does not transfer to the rested leg (Miller et al., 2005). Taken together, these findings suggest that the contralateral effect on strength is unlikely to be predominantly due to muscular adaptations, as neural mechanisms such as reduced interhemispheric inhibition are considered to mediate the cross‐education effect (Manca et al., 2021).

This study showed that the primary adaptations occurred in the ES leg; however, modest transcriptional changes occurred in the contralateral non‐ES leg. This suggests that some degree of molecular cross‐talk between the ES and non‐ES legs cannot be entirely ruled out. The upregulation of certain genes related to immune responses and developmental processes in the non‐ES leg points to the possibility that circulating factors, such as cytokines or myokines (Jameson et al., 2022; Mathers et al., 2012), may exert a minor influence on the contralateral muscle, potentially priming it for future activity or adaptation. Indeed, cyclic compressive loading has been shown to increase protein synthesis associated with mechanosensitive signalling during muscle disuse (Lawrence et al., 2020) and a period of recovery from atrophy (Miller et al., 2018). In this study, there was evidence of upregulation in several genes that regulate muscle metabolism in the non‐ES leg, such as *Tbc1d1* – which facilitates glucose transporter type 4 (GLUT4) translocation to enhance glucose uptake (An et al., 2010) – and *Mss51*, a regulator of mitochondrial protein synthesis essential for oxidative phosphorylation (Fujita et al., 2018). Among the genes that were downregulated in the non‐ES leg, *Ddit4* (also named REDD1), a repressor of mammalian/mechanistic target of rapamycin complex 1, is reported to be repressed by ES (Gordon et al., 2014). The stress responsive protein *Gadd45g* is also shown to be one of the top five genes downregulated by resistance exercise in human skeletal muscle (Pillon et al., 2020). Our findings of altered expression of these exercise responsive genes in the non‐ES leg suggest that despite the limited changes in comparison with the ES leg, the potential for subtle systemic adaptations to contribute to cross‐education effects requires further investigation at the protein level with functional assessment, particularly for long‐term training and rehabilitation settings. However, it remains to be examined whether the observed changes in the non‐ES leg are truly systemic or localised adaptations tied to spinal or cortical circuits. Also, it should be noted that more variable genes were identified after ES compared with the non‐ES group than with the control group, which may be influenced by whether the samples were taken from the same individuals or not. Although the present study was an exploratory investigation with a small number of animals, future research requires the inclusion of additional distant heterogeneous muscles (e.g. contralateral forelimb).

### 4.1 Conclusion

In conclusion, while unilateral ES induces significant transcriptional changes in the stimulated muscle, its impact on the contralateral, non‐stimulated muscle appears to be minimal. This reinforces the role of localised muscle adaptation as the primary outcome of ES and suggests that unilateral models remain valid for studying the direct effects of ES on skeletal muscle, with limited contralateral effects.

## AUTHOR CONTRIBUTIONS

Takanaga Shirai and Yu Kitaoka conceived and designed this project; Takanaga Shirai, Kazuki Uemichi, and Yu Kitaoka performed experiment; Takanaga Shirai and Yu Kitaoka analysed data; Takanaga Shirai and Yu Kitaoka interpreted results of experiments; Takanaga Shirai prepared figures; Takanaga Shirai and Yu Kitaoka drafted manuscript; Takanaga Shirai, Tohru Takemasa and Yu Kitaoka edited and revised manuscripts. All authors have read and approved the final version of this manuscript and agree to be accountable for all aspects of the work in ensuring that questions related to the accuracy or integrity of any part of the work are appropriately investigated and resolved. All persons designated as authors qualify for authorship, and all those who qualify for authorship are listed.

## CONFLICT OF INTEREST

None declared.

## Supporting information

Supporting information

## Data Availability

Raw data from this study are available from the corresponding author upon reasonable request.
