# Supplementary material for: Unilateral electrical stimulation of mice induces transcriptional response in stimulated leg with limited effect on non‐stimulated contralateral leg
Source: Exp Physiol. 2025 Feb 27;110(9):1197–202. doi: 10.1113/EP092394 (PMC12400828; doi:10.1113/EP092394)
Supplement: Supplementary file 1 — Supporting information [file EPH-110-1197-s001.pdf]

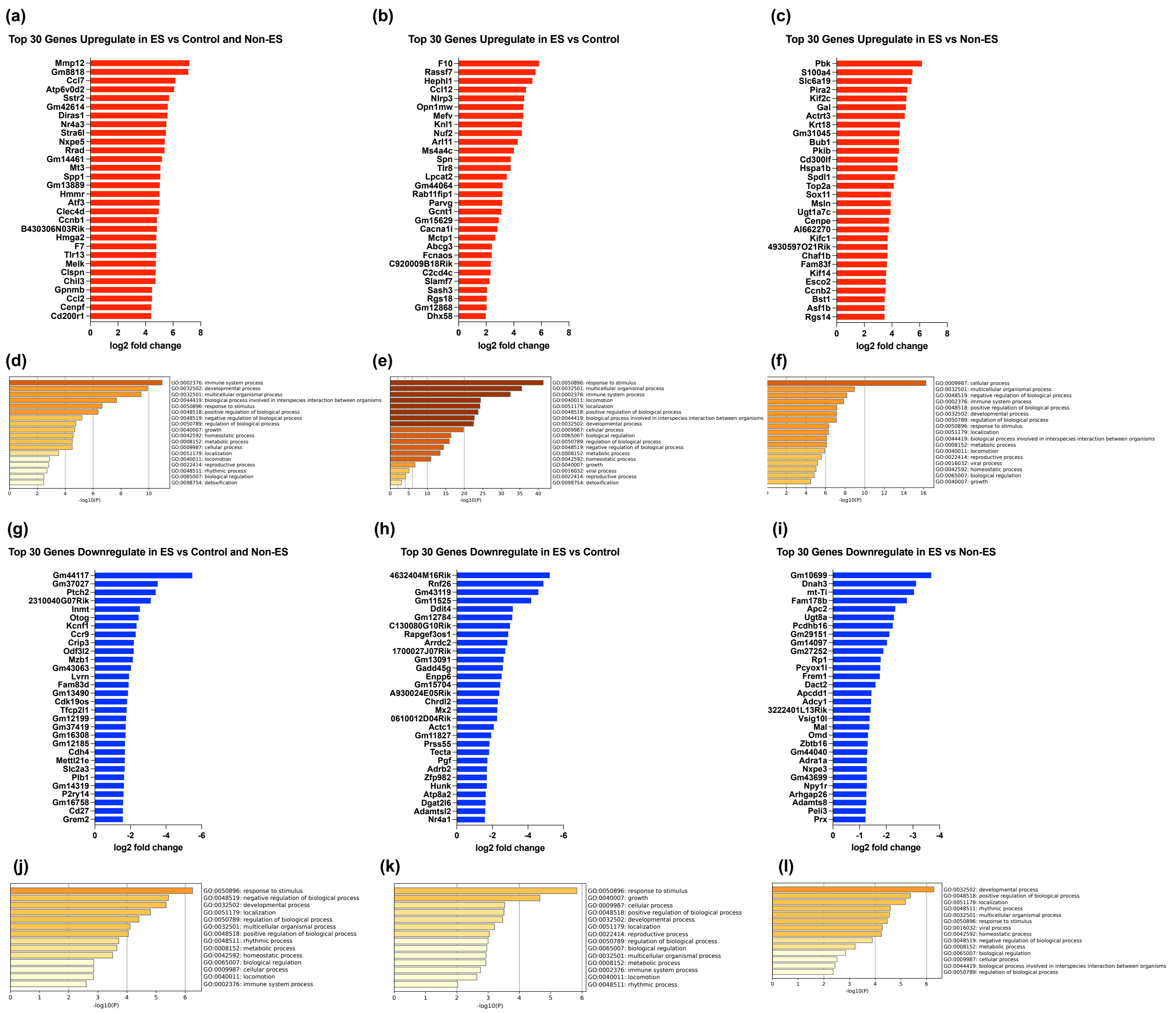

Supplementary Figure S1. Differential gene expression and GO enrichment analysis. (a-c) Top 30 upregulated genes in (a) ES versus control and non-ES, (b) ES versus control, (c) ES versus non-ES. (d-f) GO enrichment analysis for upregulated genes in (d) ES versus control and non-ES, (e) ES versus control, (f) ES versus non-ES. (g-i) Top 30 downregulated genes in (g) ES versus control and non-ES, (h) ES versus control, (i) ES versus non-ES. (j-l) GO enrichment analysis for downregulated genes in (j) ES versus control and non-ES, (k) ES versus control, (l) ES versus non-ES.
